# First Evidence of Pharmaceutical Residues in the Cerrón Grande Reservoir, El Salvador

**DOI:** 10.3390/molecules31030455

**Published:** 2026-01-28

**Authors:** Irene Romero-Alfano, Violeta Martínez, Nathaly Peña, Kevin Martínez, Carlos Castro, Maryory Velado, Oscar Carpio, Cristian Gómez-Canela

**Affiliations:** 1 Department of Analytical and Applied Chemistry, School of Engineering, Institut Químic de Sarrià, Universitat Ramon Llull, Via Augusta 390, 08017 Barcelona, Spain; ireneromeroa@iqs.url.edu; 2 Department of Process Engineering and Environmental Sciences, Universidad Centroamericana José Simeón Cañas, La Libertad 05001, El Salvador; vamartinez@uca.edu.sv (V.M.); 00007818@uca.edu.sv (N.P.); 00125419@uca.edu.sv (K.M.); 00078119@uca.edu.sv (C.C.); mvelado@uca.edu.sv (M.V.); ocarpio@uca.edu.sv (O.C.)

**Keywords:** Cerrón Grande Dam, direct injection, drugs, drug residues, risk assessment

## Abstract

This study presents a comprehensive evaluation and environmental risk assessment (ERA) of pharmaceutical residues in the Cerrón Grande Reservoir, one of the most important surface water bodies in El Salvador. Sampling campaigns were conducted over a one-year period, covering both the dry (January 2024) and rainy (July 2024) seasons. A total of 76 pharmaceutical compounds were analyzed using liquid chromatography-tandem mass spectrometry (LC-MS/MS), of which only five were not detected. During the dry season, the highest environmental concentrations were observed for mecamylamine (1710–6913 µg L^−1^), 1,7-dimethylxanthine (379–2829 µg L^−1^), chloroquine (2.29–362.7 µg L^−1^), and hydroxychloroquine (5.02–315.4 µg L^−1^). Concentrations generally decreased in the rainy season, with mecamylamine (1526–2198 µg L^−1^), 1,7-dimethylxanthine (0.018–0.55 µg L^−1^), and caffeine (0.2–0.474 µg L^−1^) remaining the most prevalent. Compounds exceeding 1 µg L^−1^ were assessed using predicted no-effect concentrations (PNEC) to calculate risk quotients (RQ). Chloroquine (RQ = 3346.3), mecamylamine (RQ = 1437.8), hydroxychloroquine (RQ = 1027.2), and manidipine (RQ = 271.0) posed the highest risks during the dry season, while only mecamylamine (RQ = 502.0) exceeded this threshold in the rainy season. To our knowledge, this represents the first in-depth study of pharmaceutical residues in Salvadoran surface waters, providing a foundational reference for future research and environmental policy in the region.

## 1. Introduction

Pharmaceutical residues are a group of contaminants of increasing environmental concern due to their widespread use, incomplete removal in wastewater treatment processes, and continuous discharge into aquatic ecosystems [1,2]. These compounds can exert toxic effects on non-target aquatic organisms, including endocrine disruption, behavioral alterations, and reproductive impairment [3,4,5,6]. Their presence in surface waters has been documented in a growing number of countries, especially in urban and peri-urban areas, where population density and medical consumption are high. However, significant geographical gaps persist, particularly in low- and middle-income countries where monitoring programs are limited or non-existent [7].

Central America represents a critical yet understudied region. Rapid urbanization, limited infrastructure for wastewater treatment, and intensive agricultural practices contribute to the potential release of pharmaceuticals and other emerging contaminants into the environment [7,8]. Despite this context, systematic studies on the presence and environmental implications of these pollutants remain scarce.

El Salvador spans an area of 21,041 km^2^ and has a population of over 6 million people. Its tropical climate features a rainy season from May to October, followed by a summer season from November to April. Although the country’s natural water network extends over 9009 km, factors such as deforestation, improper land use, erosion, and inadequate wastewater management have led to a precarious situation for its water resources [9]. The Cerrón Grande Reservoir, also known as Lake Suchitlán, is the largest artificial freshwater body in El Salvador and one of the most ecologically and socioeconomically significant. This reservoir is fed by the waters of the Lempa River and spans 607 km^2^, including its aquatic surface and several associated micro-watersheds. Within its basin, 36 rivers and streams have been identified as directly flowing into the reservoir, distributed across 23 sub-watersheds. The wetland supports a wide range of ecosystem services, including drinking water supply, fisheries, irrigation, tourism, and hydroelectric power generation [10].

The Cerrón Grande Reservoir has been identified as one of the most polluted water bodies in El Salvador, receiving significant industrial waste from sugar mills, metal industries, textile and paint factories, and paper mills, among others. Additionally, the reservoir is heavily impacted by organic waste generated nationwide, including a monthly discharge of more than 3.86 million tons of excreta from the Acelhuate River, which drains wastewater from 32 sewage systems serving over 1.5 million households in the metropolitan area of San Salvador, the capital city [11].

Shared by Guatemala, El Salvador, and Honduras, the Lempa River that originates the Cerrón Grande Reservoir covers a total area of 17,935.50 km^2^, with El Salvador accounting for 58.7% of this area. The basin’s importance relies on its provision of approximately 72% of the country’s total water resources, directly supporting 60% of the Salvadoran population [12,13]. According to data from the Ministry of the Environment and Natural Resources [14], 950 untreated wastewater discharge points have been identified in the Lempa River basin. Additionally, a study conducted in 2024 showed that only 5% of the wastewater generated in the country is treated before discharging into a receiving body [11]. Despite the extensive documentation of water pollution in the Lempa River, there are few detailed studies on the presence of emerging contaminants (ECs) in Salvadoran water sources.

ECs encompass a wide range of natural and anthropogenic substances found in water at low concentrations, ranging from nanograms per liter (ng/L) to micrograms per liter (µg/L) [15]. This group includes pharmaceuticals and personal care products (PPCPs), industrial chemicals, pesticides, steroid hormones and endocrine-disrupting compounds (EDC), and disinfection by-products (DBP) [16,17,18]. While their effects on the daily activities and internal biology of organisms may go initially unnoticed, prolonged exposure may lead to significant cellular imbalance [15,19].

The management and control of EC have posed a significant challenge for many governments worldwide. The European Union (EU) has implemented The Watch List, which tracks various selected substances to identify and prioritize those that may pose negative effects on the environment and human health [20], including pharmaceuticals along with their predicted no-effect concentrations [21]. Switzerland has proposed a similar approach, adopting environmental quality standards for water quality criteria related to pharmaceutical compounds such as azithromycin and diclofenac, as well as pesticides like diazinon and cypermethrin [19].

In El Salvador, the only study on the presence of these species in water identified 16 EC in 11 samples collected from the Lempa River basin, with sulfamethoxazole (23 µg/L), sucralose (6 µg/L), and bisphenol A (2 µg/L) as the most predominant species [22].

This study outlines the first investigation into the occurrence and environmental risk of pharmaceutical residues in the Cerrón Grande Reservoir. Using direct injection analysis followed by liquid chromatography–mass spectrometry (LC-MS/MS), we analyzed water samples collected from multiple sites within the reservoir to identify and quantify a selection of commonly used pharmaceuticals, including antibiotics, analgesics, and anti-inflammatory drugs. To complement the occurrence data, we performed a preliminary environmental risk assessment (ERA) by comparing the measured environmental concentrations (MECs) with predicted no-effect concentrations (PNECs) for various aquatic organisms. This approach allowed us to estimate risk quotients (RQs) and identify compounds of potential ecological concern. The findings of this study provide crucial baseline data on the occurrence of pharmaceutical contamination in one of El Salvador’s key aquatic ecosystems and contribute to the broader understanding of emerging contaminants in tropical freshwater environments. By performing the first ERA and estimating the RQs of the pharmaceuticals residues in the reservoir, the results also underscore the need for the implementation of monitoring strategies and pollution mitigation measures to preserve water quality and protect aquatic life in El Salvador and other vulnerable water bodies across the region. The specific goals of this study are to: (i) assess the presence of selected pharmaceuticals in the Cerrón Grande Dam, (ii) perform an ERA and estimate the RQs of the compounds detected on each site, and (iii) evaluate the influence of seasonal variation.

## 2. Results

### 2.1. Quality Assurance

Quality assurance parameters demonstrated a strong analytical performance. Detailed values for each parameter are found in Table S1. The calibration curves demonstrated a good linearity across the concentration range tested, while limits of detection (LOD) were low enough for the trace-level quantification. Lastly, recovery and matrix effect obtained values remained within acceptable ranges.

### 2.2. Pharmaceutical Residues in Cerrón Grande Reservoir

**Figure 1:**
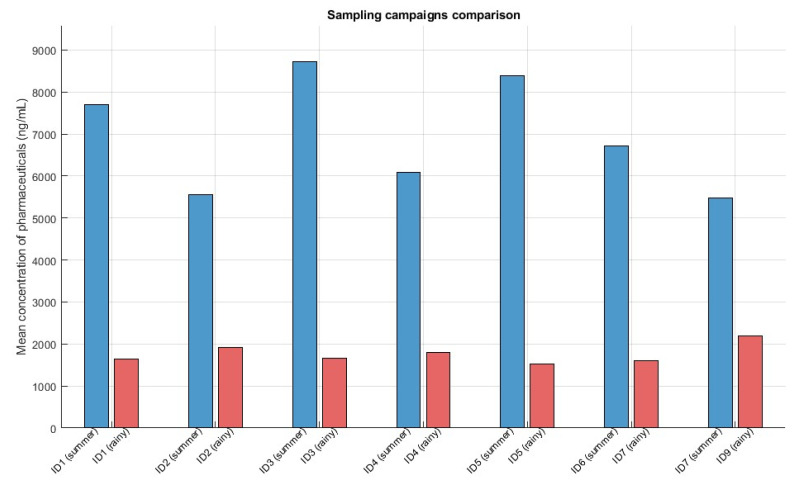
Seasonal comparison of pharmaceutical mean concentrations across equivalent sampling locations in the Cerrón Grande reservoir.

Concentrations of pharmaceuticals were calculated in the Cerrón Grande Reservoir at 12 sampling points during the summer season, and 9 sampling points during the rainy season. Out of 76 pharmaceuticals studied, only five were not detected. The rest of them were detected in more than 50% of the samples analyzed, in the orders of μg L^−1^. Table S2 shows the levels of pharmaceuticals in the 12 sampling points in the summer season. The concentrations in this first sampling campaign ranged 0.00058 and 6913 µg L^−1^. The pharmaceutical with highest concentrations was mecamylamine, a non-selective, non-competitive antagonist of the nicotinic acetylcholine receptors (nAChRs) that was introduced in the 1950s as an antihypertensive drug. This drug was detected in all sampling points of the summer season at concentrations between 1710 and 6913 µg L^−1^. Table 1 summarizes the minimum, maximum and mean values of all target compounds in each sampling campaign. Mecamylamine was also detected at high concentrations in the rainy season, with a maximum level of 2198 µg L^−1^ in the ID9 sampling point (Table 1). To the authors knowledge, this is the first time that this drug is monitored in surface water. Also, Figure 1 illustrates the seasonal variation in the mean concentrations of the target pharmaceuticals across equivalent sampling locations within the Cerrón Grande reservoir. This comparison highlights temporal fluctuations associated with wet and dry periods, allowing the assessment of potential influences of hydrological dynamics and seasonal loading on contaminant distribution patterns.

On the other hand, 1,7-dimethylxanthine was also detected in the samples. This compound is an isomer of theophylline and theobromine, two well-known stimulants found in coffee, tea, and chocolate, mainly in the form of caffeine. The levels of this pharmaceutical ranged from 379 to 2829 µg L^−1^ in the summer season (Table 1). However, the levels decreased considerably in the rainy season campaign (0.018–0.55 µg L^−1^). In 2021, Ramírez-Morales et al. (2021) reported levels of pharmaceuticals between 0.013 μg L^−1^ and 53.8 μg L^−1^ in surface waters, being caffeine, 1,7-dimethylxanthine, ofloxacin, gemfibrozil and cephalexin the most concentrated drugs [23]. The next pharmaceuticals were chloroquine and hydroxychloroquine, two antimalarial drugs that are effective against certain types of malaria parasites, including *Plasmodium vivax*, *P. ovale*, and *P. malariae*. Moreover, both drugs were explored early in the COVID-19 pandemic due to their known antiviral and immunomodulatory effects. Initial laboratory studies showed some antiviral activity, but clinical trials did not confirm meaningful benefits. As a result, major health authorities such as the WHO, FDA, and EMA no longer recommend chloroquine or hydroxychloroquine for the treatment or prevention of COVID-19, due to lack of efficacy and potential side effects. In the present study, chloroquine was detected in the range of 2.29 to 362.7 µg L^−1^ in the summer season. However, the levels of this drug were below detection limit in the rainy season campaign (Table S3). On the other hand, hydroxychloroquine was detected in the range of 5.02 to 315.4 µg L^−1^ in the summer season, and the levels were also below the limit of detection in the rainy season campaign (Table S3).

Valsartan, a widely used pharmaceutical used to treat high blood pressure, heart failure, and diabetic kidney disease was detected as mean concentration of 43.3 µg L^−1^ in the summer season, with ID10 and ID11 as the most contaminated sampling points with 81.6 and 86.3 µg L^−1^, respectively (Table S2). However, in the rainy season, the levels were much lower with levels in the range of 0.0139 to 0.0721 µg L^−1^ (Table S3). Valsartan has been detected in other studies at concentrations of 0.648 µg L^−1^ in surface water [24]. Acetaminophen was also detected in all the samples analyzed both in the summer season campaign and in the rainy season, being the mean concentration 34.5 and 0.030 µg L^−1^, respectively (Table 1). Acetaminophen is the most detected drug in surface waters, and it has been studied a lot in recent years [25,26]. On the other hand, norfloxacin was detected in the range of 0.784 and 68.3 µg L^−1^ in the summer season and decreased considerably in the rainy season (0.002 to 0.004 µg L^−1^) [27].

The other pharmaceuticals studied were detected between 0.001 (sulfadiazine) and 50.4 µg L^−1^ (caffeine) in the summer season [28,29]. However, target compounds were detected between 5e^−5^ (scopolamine) and 0.474 µg L^−1^ (caffeine) in the rainy season sampling campaign [30].

The elevated concentrations observed for certain compounds may be influenced by local conditions within the Cerrón Grande Reservoir watershed. The reservoir receives continuous input from densely populated areas where wastewater treatment coverage is limited, resulting in the direct discharge of untreated or partially treated effluents. In addition, agricultural and livestock activities in the surrounding region may contribute further loads of pharmaceuticals and metabolites. These combined pressures create a context in which environmental concentrations can exceed those typically reported in regions with more advanced wastewater management systems.

**Figure 2:**
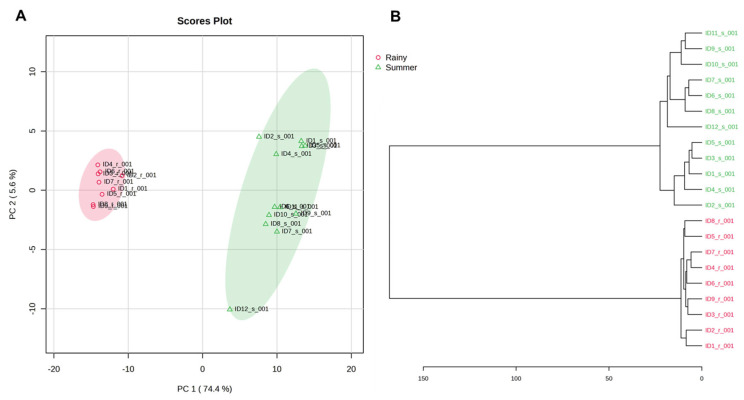
Multivariate analysis of pharmaceutical residues in the Cerrón Grande Reservoir based on concentrations from both sampling campaigns: (**A**) principal component analysis (PCA) showing seasonal differentiation between dry (green triangles) and rainy periods (red circles), and (**B**) hierarchical cluster analysis (dendrogram) illustrating sample grouping according to seasonal patterns.

To further explore the distribution of samples from both sampling campaigns, a principal component analysis (PCA) (Figure 2A) and hierarchical cluster analysis (dendrogram) (Figure 2B) were performed using concentration values to assess potential seasonal patterns in pharmaceutical occurrence.

The PCA score plot (Figure 2A) shows a clear separation between samples collected during the dry and rainy seasons along PC1, which explains 74.4% of the total variance, indicating that seasonality is the main factor influencing the distribution of pharmaceutical residues in the Cerrón Grande Reservoir. Dry season samples form a more compact cluster, suggesting relatively homogeneous concentration profiles, whereas rainy season samples display greater dispersion, likely due to dilution effects and variable runoff during periods of increased precipitation.

These patterns are corroborated by the hierarchical cluster analysis (Figure 2B), which distinctly groups samples according to sampling season rather than spatial location, highlighting the dominant role of temporal variability in shaping pharmaceutical occurrence in the reservoir.

### 2.3. Pharmaceuticals Prioritization

The Environmental Risk Assessment (ERA) prioritized pharmaceuticals based on both occurrence data and concentration thresholds that indicated potential environmental relevance. In the ERA, compounds were chosen if their measured concentrations exceeded 1 µg L^−1^ in at least one sampling campaign, regardless of whether it was dry or rainy seasons.

This approach ensures that the evaluation focuses on pharmaceuticals with the greatest likelihood of showing measurable biological effects, either through chronic exposure or bioaccumulation. This concentration criterion allows the analysis to focus on substances that have higher persistence, usage frequency, or resistance to conventional wastewater treatment processes.

Based on these criteria, the pharmaceuticals included in the ERA, listed in descending order of concentration, were: 1,7-dimethylxanthine, 4-acetamidoantipyrine, amitriptyline, bicalutamide, bisoprolol, caffeine, chloroquine, cimetidine, fexofenadine, fluoxetine, fluvoxamine, hydroxychloroquine, ivermectin, levofloxacin, lidocaine, manidipine, mecamylamine, mevinolin, norfloxacin, oxytetracycline, zuclopenthixol, acetaminophen, metformin, sulfamethoxazole, acyclovir, sarafloxacin, furosemide, topiramate, and valsartan.

These compounds represent a wide variety of therapeutic classes, such as antibiotics, antivirals, antihypertensives, antidepressants, and antimalarials, and they show different usage patterns and pharmacological properties. Their detection at concentrations above 1 µg L^−1^ suggests potential persistence in the aquatic environment.

**Figure 3:**
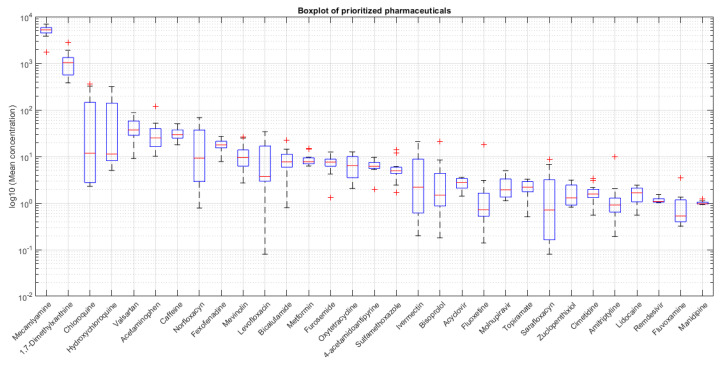
Concentration distribution of prioritized pharmaceuticals in the Cerrón Grande reservoir during summer season. Mean concentrations are plotted on a logarithmic scale (log_10_) to represent the wide concentration range.

Figure 3 provides an overview of the seasonal variability and supports the selection of priority substances for risk assessment by summarizing the average concentrations of every compound across both sampling campaigns.

### 2.4. Risk Assessment

Risk assessment was calculated through the determination of the Risk Quotient (RQ), which expresses the ratio of the Measured Environmental Concentration (MEC) (Table 1) of the prioritized compounds to the Predicted No Effect Concentration (PNEC) [31,32]. This evaluation allows for the identification of potential threats that the presence of the detected pharmaceuticals may pose to aquatic species. According to various studies, RQ < 1 indicates no significant risk; values between 1 ≤ RQ < 10 indicate a low risk of adverse effects; values between 10 ≤ RQ < 100 suggest a potential for adverse effects; and finally, RQ ≥ 100 indicates a high potential for adverse effects [31,32,33,34]. The RQ results for the priority compounds, along with the PNEC values used for calculation, are shown in Table 2. Additionally, toxicologically relevant concentrations (EC_50_ and LC_50_) determined for some compounds are included, indicating the organisms used for their measurement.

Of the 31 compounds analyzed, chloroquine (RQ = 3346.3), mecamylamine (RQ = 1437.8), hydroxychloroquine (RQ = 1027.2), and manidipine (RQ = 271.0) exhibited an average RQ ≥ 100 during the summer season, whereas in the rainy season, only mecamylamine (RQ = 502.0) reached this threshold. Previous studies have reported chloroquine RQ values ranging from 0.3 to 9.77 in surface waters in China [35], and average RQ values below 1 for both chloroquine and hydroxychloroquine in surface waters in Spain [32]. No additional reports were found for mecamylamine and manidipine. The values detected in the Cerrón Grande Reservoir are therefore significantly higher than those reported in these countries, clearly indicating a high potential risk to aquatic species. In the second risk category (10 ≤ RQ < 100), the compounds included mevinolin (RQ = 60), 1,7-dimethylxanthine (RQ = 52.7), norfloxacin (RQ = 40.2), ivermectin (RQ = 40), levofloxacin (RQ = 38.7), caffeine (RQ = 26.4), fluoxetine (RQ = 24.5), zuclopenthixol (RQ = 20), and amitriptyline (RQ = 12). During the rainy season, only manidipine (RQ = 16.3) fell within this category. The marked reduction in RQ values during the rainy season may be attributed to increased water levels in the wetland, which likely diluted the concentrations of these compounds; nevertheless, further research is needed to better understand and correlate the influence of seasonal variations.

However, it is important to consider the potential variability associated with the use of different PNEC values reported in the literature and with the assessment factors applied during their derivation. Such variability may influence the magnitude of the calculated RQs and, consequently, the interpretation of risk levels, particularly for compounds classified as high-risk (RQ ≥ 100). This contextualization provides a more robust framework for understanding the sensitivity of the results discussed above.

While the RQ calculations provide quantitative risk thresholds, the actual ecological impacts may be substantially modulated by compound- and species-specific uptake mechanisms, with studies demonstrating that bioaccumulation factors can vary by up to 110-fold among different fish species for certain pharmaceuticals such as antihistamines [55]. Resident fish populations may be subjected to chronic low-level exposure, even when acute toxicity thresholds are not exceeded, due to the continuous input of pharmaceutical residues creating a condition of pseudopersistence in the reservoir. Furthermore, mixture toxicity effects following the concentration addition (CA) principle indicate that combined RQ values calculated under single-chemical scenarios may substantially underestimate actual ecological risks [56].

## 3. Experimental Section

### 3.1. Chemicals and Reagents

#### 3.1.1. Chemicals

Seventy standards were purchased from Sigma-Aldrich (St. Louis, MO, USA), three from BLD Pharm (Shanghai, China) and three from Cayman Chemicals Company (Ann Arbor, MI, USA). Table S1 shows the CAS numbers of all target compounds. Purity values of the standard bought ranged between 98–99%. On the other hand, HPLC grade acetonitrile (ACN), methanol (MeOH) and formic acid (HCOOH) were supplied by Fisher Scientific Chemical (Bridgewater, MA, USA). Finally, ultra-pure Milli-Q water was daily obtained from Millipore Q-POD (0.22 μm) filtration and purification system (Millipore, Bedford, MA, USA).

#### 3.1.2. Standard Preparations

Stock solutions were prepared at a concentration of 1000 ng µL^−1^ in methanol. Working solutions were subsequently diluted to 5 ng µL^−1^ using a 90:10 (*v*/*v*) mixture of Milli-Q water and methanol. All solutions were stored in the dark at −20 °C to avoid possible degradations.

### 3.2. Sampling Campaigns

**Figure 4:**
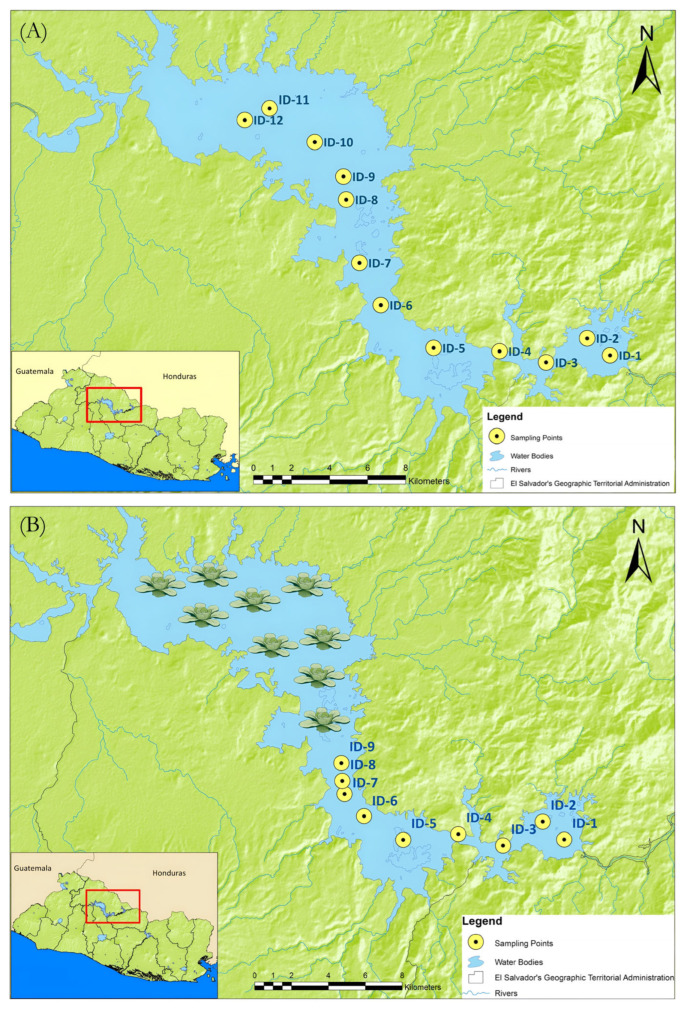
Sampling locations within the reservoir during two distinct seasons: (**A**) summer season and (**B**) rainy season. The maps illustrate the spatial distribution of sampling points used for water quality assessment in each period.

Grab samples were collected in the Cerrón Grande Reservoir at 12 locations during the summer season (January 2024) and 9 locations during the rainy season (July 2024). Figure 4 shows all sampling points in the summer and rainy season sampling campaigns. A total volume of 3 L was collected at each site in polyethylene containers. Sampling was conducted on the surface at a depth of less than 1 m by directly filling the bottle with water. Before collecting the sample, the bottle was rinsed three times with water from the reservoir to adapt it to field conditions. During the rainy season, it was inviable to access the upper part of the reservoir to reach sampling points ID8–ID12 from summer season (Figure 4A) due to the proliferation of aquatic plants (*Pistia stratiotes*). This proliferation, along with the presence of algae, has been reported annually in other studies and is attributed to the proximity of the area to the confluence of the Lempa River and the Acelhuate River [13,57,58,59,60,61,62,63,64], the latter being the most polluted river in El Salvador. Therefore, the number of sampling points was reduced during the rainy season (Figure 4B).

### 3.3. Sample Pretreatment and LC-MS/MS Analysis

After water collection, samples were stored in dark conditions at 4 °C. Before used, the water was filtered through a 0.22 µm Nylon filters (Phenomenex, Torrance, CA, USA) into chromatographic vials. Then, the identification and quantification of environmental samples were performed using liquid chromatography coupled to tandem mass spectrometry (LC-MS/MS), based on previously established methodologies [65] optimized in a Exion LCTM liquid chromatograph coupled to a triple quadrupole mass spectrometer (Triple Quad 7500 System-QTrap^®^ Ready, SCIEX, Framingham, MA, USA) equipped with an electrospray ionization source (ESI). Chromatographic separation was achieved on a Kinetex Polar C18 column (150 × 2.1 mm, 2.6 µm, 100 Å) (Phenomenex, Torrance, CA, USA). The mobile phases consisted on Milli-Q water with 0.1% formic acid (aqueous phase, A) and acetonitrile with 0.1% formic acid (organic phase, B). A gradient elution was applied, starting at 5% B with a flow rate of 300 µL min^−1^, for the analysis of 100 µL of each sample, following conditions previously described in the literature.

Regarding the MS/MS method, all targeted compounds were measured under positive ionization (ESI+) conditions using a Multiple Reaction Monitoring (MRM) method previously optimized in the bibliography [65] which contained a precursor ion for each compound, as well as two transitions (product ions) specifically for them. The data obtained was processed using SCIEX OS (version 3.4.5.828) software.

Representative chromatograms from the standards mix (1 µg L^−1^) (Figure S1) and the samples (Figure S2) analyzed can be found in the Supplementary Materials.

### 3.4. Quality Parameters

The main validation criteria were adopted from the previous study’s approach [65] to quality assessment. Linearity was evaluated across 0.001 and 5 µg L^−1^. Repetitive injections were used to assess the intra-day precision, and recoveries were determined in both ultra-pure and real water matrices using the next equation:

$$R\;\left(\%\right)=\;\frac{C_{\mathrm{Sample}}-C_{\mathrm{Blank}}}{C_{\mathrm{Theoretical}}}\;\times 100$$

where C_Sample_ is the concentration of each analyte in the spiked water samples, C_Blank_ is the concentration of each analyte and C_Theoretical_ is the spiked theoretical concentration of each analyte.

To distinguish between ion suppression and enhancement, matrix effects were calculated using the following equation:

$$ME\;\left(\%\right)=\;\frac{A-B}{C}\;\times 100$$

where A represents the area of each pharmaceutical in water sample, B represents the area of each compound in non-spiked waters, and C represents the area of each pharmaceutical in the calibration curve at the same concentration as the spiked sample.

Finally, signal-to-noise ratios of 3 and 10 were used to define LODs and LOQs, respectively.

### 3.5. Statistical Analysis

Data processing was performed using MATLAB (version R2024b, MathWorks, Natick, MA, USA). Descriptive graphics were used to summarize quantitative variables, expressed as mean ± standard deviation or median and interquartile range, depending on the data distribution. Graphical representation of the results was also performed using MATLAB’s built-in data visualization tools, enabling clear and accurate interpretation of the findings.

Statistical analysis was conducted using Metaboanalyst 6.0 platform to perform a Partial Least Squares (PCA) analysis and a hierarchical clustering dendrogram, in order to see the distribution of the collected data.

## 4. Conclusions

This study represents the first effort to quantify the prevalence and assess the environmental risk of pharmaceutical residues in the Cerrón Grande Reservoir, one of the most significant surface water sources in El Salvador. Findings reveal that, out of the 76 compounds evaluated, only five were not detected. During the dry season, the pharmaceuticals with the highest concentrations were mecamylamine, 1,7-dimethylxanthine, chloroquine, and hydroxychloroquine. In the rainy season, all detected concentrations decreased, likely attributable to increased river flow feeding the reservoir. Nevertheless, the compounds with the highest occurrence during this period were mecamylamine, 1,7-dimethylxanthine, and caffeine. Overall, the concentrations observed in this study are significantly higher than those reported in other countries for pharmaceutical contamination in surface waters. Environmental risk assessment of the 31 compounds with concentrations exceeding 1 µg L^−1^ revealed that chloroquine, mecamylamine, hydroxychloroquine, and manidipine exhibited risk quotient (RQ) values greater than 100, indicating a high potential for adverse ecological effects. The findings of this study represent an important baseline on the prevalence of pharmaceutical residues and their associated environmental risk in one of El Salvador’s key aquatic ecosystems, thus contributing to the broader understanding of emerging contaminants in vulnerable regions and promoting informed regional water management and regulatory frameworks.

## Figures and Tables

**Table 1:** Pharmaceuticals minimum, maximum, and mean values in each sampling campaign. Data is presented in µg L^−1^. <LOD: below detection limit.

Pharmaceuticals	Summer Season Campaign			Rainy Season Campaign		
	Min	Max	Mean	Min	Max	Mean
Mecamylamine	1711	6913	5061	1526	2198	1767
1,7-Dimethylxanthine	380	2829	1129	0.018	0.555	0.322
Chloroquine	2.29	363	87	<LOD	<LOD	<LOD
Hydroxychloroquine	5.02	315	72.9	<LOD	<LOD	<LOD
Valsartan	9.01	86.3	43.3	0.014	0.072	0.032
Acetaminophen	10.2	120.6	34.5	0.009	0.048	0.03
Caffeine	17.8	50.4	31.6	0.2	0.474	0.286
Norfloxacin	0.784	68.3	21.9	0.002	0.004	0.003
Fexofenadine	7.8	27.1	17.9	0.002	0.008	0.005
Mevinolin	2.69	26.3	11.4	0.03	0.076	0.051
Levofloxacin	0.08	34.2	9.67	<LOD	0.002	0.002
Bicalutamide	0.799	22.6	8.96	<LOD	0.003	0.003
Metformin	6.22	14.8	8.86	0.14	0.142	0.142
Furosemide	1.31	12.5	7.42	0.01	0.022	0.014
Oxytetracycline	2.05	12.5	6.55	0.062	0.218	0.116
4-acetamidoantipyrine	1.97	9.62	6.34	0.022	0.026	0.025
Sulfamethoxazole	1.69	14.1	5.88	0.03	0.038	0.033
Ivermectin	0.198	20.9	5.6	<LOD	<LOD	<LOD
Bisoprolol	0.184	21	3.84	<LOD	<LOD	<LOD
Acyclovir	1.41	3.57	2.68	0.006	0.015	0.011
Fluoxetine	0.136	18.1	2.45	<LOD	<LOD	<LOD
Molnupiravir	1.13	4.95	2.37	0.08	0.083	0.082
Topiramate	0.51	3.26	2.22	0.005	0.028	0.014
Sarafloxacyn	0.082	8.57	2.17	<LOD	<LOD	<LOD
Zuclopenthixol	0.819	3.1	1.72	0.079	0.08	0.079
Cimetidine	0.55	3.35	1.71	0.018	0.029	0.023
Amitriptyline	0.195	9.89	1.68	<LOD	<LOD	<LOD
Lidocaine	0.549	2.42	1.56	0.005	0.007	0.006
Remdesivir	1.02	1.53	1.15	0.1	0.101	0.101
Fluvoxamine	0.324	3.43	1.02	0.003	0.005	0.004
Manidipine	0.919	1.23	1.01	0.091	0.091	0.091
Diclofenac	0.324	2.95	0.982	<LOD	0.008	0.008
Tetracycline	0.293	2.6	0.973	0.023	0.027	0.024
Desloratadine	0.211	3.13	0.95	0.015	0.015	0.015
Verapamil	0.088	4.23	0.819	0.006	0.006	0.006
Citalopram	0.113	1.76	0.774	<LOD	<LOD	<LOD
Lamotrigine	0.243	0.95	0.722	0.004	0.006	0.005
Omeprazole	0.612	0.794	0.717	0.06	0.062	0.061
Trimethoprim	0.474	1.16	0.698	0.002	0.003	0.003
Rotigotine	0.13	0.901	0.604	0.003	0.013	0.008
Metoprolol	0.199	1.36	0.554	<LOD	0.006	0.006
Amiodarone	0.278	2.69	0.53	0.027	0.028	0.027
Venlafaxine	0.192	1.1	0.46	0.014	0.016	0.015
Vildagliptin	0.319	0.686	0.453	0.003	0.006	0.004
Levetiracetam	0.168	0.665	0.384	0.003	0.004	0.004
Nortriptyline	0.012	1.19	0.353	<LOD	<LOD	<LOD
Lopinavir	0.046	1	0.352	0.002	<LOD	<LOD
Quetiapine	0.031	1.36	0.348	<LOD	0.004	0.0025
Ifosfamide	0.097	0.723	0.308	0.015	0.032	0.023
4-aminoantipyrine	0.305	0.305	0.305	<LOD	<LOD	<LOD
Antipyrine	0.205	0.362	0.305	0.003	0.005	0.004
Chlormethiazole	0.155	0.557	0.219	0.016	0.019	0.017
Ritonavir	0.025	0.743	0.218	<LOD	0.002	0.002
Fenofibrate	0.016	1.27	0.213	0.002	0.003	0.0025
Scopolamine	0.022	0.695	0.202	<LOD	0.002	0.002
Capecitabine	0.043	0.364	0.163	<LOD	0.003	0.003
Rosuvastatin	0.085	0.375	0.143	0.008	0.008	0.008
Levetiracetam	0.078	0.264	0.142	0.003	0.005	0.004
Chlorpromazine	0.038	0.305	0.136	0.003	0.003	0.003
Sitagliptin	0.048	0.239	0.128	<LOD	0.003	0.003
Ranolazine	0.069	0.17	0.106	<LOD	0.002	0.002
Diflubenzuron	0.017	0.705	0.102	<LOD	<LOD	<LOD
Propranolol	0.022	0.317	0.096	<LOD	<LOD	<LOD
Reboxetine	0.006	0.316	0.094	<LOD	0.003	0.002
Atorvastatin	0.045	0.091	0.068	0.004	0.005	0.004
Clopidogrel	0.008	0.081	0.042	0.003	0.004	0.0035
Sulphapyridine	0.009	0.114	0.037	<LOD	<LOD	<LOD
Trazodone	0.006	0.075	0.036	<LOD	<LOD	<LOD
Pentoxifylline	0.012	0.039	0.025	<LOD	<LOD	<LOD
Cyclophosphamide	0.009	0.048	0.023	<LOD	<LOD	<LOD
Gliclazide	0.011	0.04	0.019	<LOD	<LOD	<LOD
Sulfadiazine	<LOD	0.014	0.014	<LOD	0.002	0.0015
Dexamethasone	<LOD	<LOD	<LOD	<LOD	0.003	0.002
Fluticasone	<LOD	<LOD	<LOD	0.002	0.002	0.002
Mycophenolic acid	<LOD	<LOD	<LOD	0.004	0.015	0.006
Ranitidine	<LOD	<LOD	<LOD	<LOD	0.004	0.004

**Table 2:** RQ, PNEC, LC50, and EC50 of the priority compounds detected in the sampling campaigns.

Acute Toxicity (µg/L)		Summer Season												Rainy Season								
Test	Data	ID1	ID2	ID3	ID4	ID5	ID6	ID7	ID8	ID9	ID10	ID11	ID12	ID1	ID2	ID3	ID4	ID5	ID6	ID7	ID8	ID9
1,7 Dimethylxanthine		24.7	26.3	44.6	26.2	47.0	50.0	52.4	50.7	132.2	72.0	89.1	17.7	<1	<1	<1	<1	<1	<1	<1	<1	<1
PNEC	21.4 [30]																					
LC_50_	>100,000 (Crustaceans) [31]																					
4-Acetamidoantipyrine		<1	<1	<1	<1	<1	<1	<1	<1	<1	<1	<1	<1	<1	<1	<1	<1	<1	<1	<1	<1	<1
PNEC	100 [30]																					
LC_50_	4690 (*Danio rerio*) [32]																					
Amitriptyline		14.5	4.1	7.8	5.2	70.6	7.0	6.0	4.8	10.2	4.4	8.1	1.4	<1	<1	NA ^a^	<1	<1	<1	NA	NA	<1
PNEC	0.14 [30]																					
LC_50_	1900 (*Danio rerio*) [33]																					
Bicalutamide		14.4	6.6	22.6	11.4	8.6	8.8	5.7	4.7	10.9	6.7	6.1	<1	<1	<1	<1	NA	<1	NA	NA	<1	<1
PNEC	1 [30]																					
LC_50_	0.0161 (Green algae) [34]																					
Bisoprolol		<1	<1	<1	<1	<1	<1	<1	<1	<1	<1	<1	<1	NA	NA	NA	NA	NA	NA	NA	NA	NA
PNEC	92 [30]																					
EC_50_	>100,000 (*Daphnia magna*) [35,36]																					
Caffeine		22.7	24.9	42.0	32.6	29.5	22.9	24.2	17.8	36.9	18.7	29.3	14.8	<1	<1	<1	<1	<1	<1	<1	<1	<1
PNEC	1.2 [30]																					
LC_50_	58.9–1703.01 (*Danio rerio*) [37]																					
Chloroquine		12,392.4	106.0	8649.7	88.0	2478.3	731.1	122.0	107.1	13,949.3	170.6	1264.5	96.9	NA	NA	NA	NA	NA	NA	NA	NA	NA
PNEC	0.026 [30]																					
LC_50_	4250 (*Proales similis*) [38]																					
Cimetidine		<1	<1	<1	<1	<1	<1	<1	<1	<1	<1	<1	<1	<1	<1	<1	<1	<1	<1	<1	<1	<1
PNEC	176 [30]																					
EC_50_	271,300–740,000 (*Dapnia magna*) [39]																					
Fexofenadine		<1	<1	<1	<1	<1	<1	<1	<1	<1	<1	<1	<1	<1	<1	<1	<1	<1	<1	<1	<1	<1
PNEC	200 [30]																					
Fluoxetine		17.0	3.3	15.2	5.5	181.0	7.5	5.6	5.0	30.6	6.8	15.5	1.4	<1	<1	<1	<1	NA	NA	<1	<1	NA
PNEC	0.1 [30]																					
LC_50_	820 (*Daphnia magna*) [40]																					
Fluvoxamine		<1	<1	<1	<1	1.4	<1	NA	NA	<1	NA	NA	NA	<1	<1	NA	<1	<1	<1	NA	NA	NA
PNEC	2.49 [30]																					
LC_50_	840 (*Ceriodaphnia dubia*) [41]																					
Hydroxychloroquine		4442.1	140.1	2671.8	125.6	1265.7	177.6	110.5	117.8	2884.9	70.8	231.2	87.8	NA	NA	NA	NA	NA	NA	NA	NA	NA
PNEC	0.071 [30]																					
LC_50_	243,012 (*Danio rerio*) [3]																					
Ivermectin		59.9	5.3	96.4	3.4	149.2	1.6	65.5	58.8	18.2	13.1	6.9	1.4	<1	<1	NA	NA	NA	NA	NA	NA	NA
PNEC	0.14 [30]																					
LC_50_	0.025 (*Daphnia magna*) [42]																					
EC_50_	440 (*Danio rerio*) [43]																					
Levofloxacin		65.7	79.2	68.1	15.5	136.6	13.3	14.1	4.9	42.8	12.0	11.5	<1	<1	<1	<1	NA	<1	<1	NA	NA	NA
PNEC	0.25 [30]																					
EC_50_	7.9 (*Microcystis aeruginosa*), 51 (*Lemna minor*) [44]																					
Lidocaine		<1	<1	<1	<1	<1	<1	<1	<1	<1	<1	<1	<1	<1	<1	<1	<1	<1	<1	<1	<1	<1
PNEC	600 [30]																					
EC_50_	308,800 (*Daphnia magna*) [45]																					
Manidipine		361.0	NA	397.0	296.5	319.1	NA	308.9	335.9	316.2	310.6	308.6	298.0	NA	29.3	29.3	NA	29.3	NA	29.3	NA	29.3
PNEC	0.0031 [30]																					
Mecamylamine		1766.5	1353.8	1964.0	1510.2	1953.0	1538.6	1183.7	1438.4	1085.8	1424.9	1549.0	486.0	464.7	544.7	473.8	510.0	433.4	507.1	456.4	503.4	624.5
PNEC	3.52 [30]																					
Mevinolin		32.6	138.2	86.2	59.4	131.1	58.4	32.7	47.1	53.3	38.4	28.5	14.1	NA	<1	NA	<1	<1	NA	<1	<1	NA
PNEC	0.19 [30]																					
Norfloxacin		136.5	18.5	132.4	24.2	68.5	6.0	5.8	1.6	75.1	4.3	9.3	NA	NA	<1	<1	<1	NA	NA	NA	NA	NA
PNEC	0.5 [30]																					
LC_50_	29,880 (*Brachionus calyciflorus*) [40]																					
EC_50_	16,600 (*Selenastrum capricornutum*) [40]																					
Oxytetracycline		13.0	12.6	6.7	8.0	22.2	NA	NA	NA	NA	4.1	25.1	NA	NA	<1	<1	<1	<1	<1	<1	NA	NA
PNEC	0.5 [30]																					
LC_50_	110,100 (*Oryzias latipes*) [40]																					
EC_50_	18,650 (*Ceriodaphnia dubia*) [40]																					
Zuclopenthixol		62.0	16.4	48.5	NA	25.7	NA	NA	NA	48.8	NA	22.1	16.8	1.6	1.6	1.6	1.6	1.6	NA	1.6	1.6	1.6
PNEC	0.05 [30]																					
Acetaminophen		1.1	<1	2.6	<1	<1	<1	<1	<1	<1	<1	<1	NA	<1	<1	<1	<1	<1	<1	<1	<1	<1
PNEC	46 [30]																					
LC_50_	5320 (*Daphnia magna*) [46]																					
EC_50_	1120 (*Daphnia magna*) [46]																					
Metformin		<1	NA	<1	<1	<1	<1	<1	<1	<1	<1	<1	<1	NA	NA	NA	NA	<1	NA	NA	NA	NA
PNEC	160 [30]																					
LC_50_	1,315,500 (*Danio rerio*) [47]																					
Sulfamethoxazole		9.3	2.8	8.3	7.9	4.0	8.1	7.1	23.5	7.3	20.1	10.3	8.9	<1	<1	<1	<1	<1	<1	<1	<1	<1
PNEC	0.6 [30]																					
LC_50_	>750,000 (*Oryzias latipes*) [31,48,49]																					
EC_50_	25,200 (*Daphnia magna*) [31,48,49]																					
Acyclovir		<1	<1	<1	<1	<1	<1	<1	<1	<1	<1	<1	<1	<1	<1	<1	<1	<1	<1	<1	<1	<1
PNEC	5.6 [30]																					
EC_50_	3062 (*Ceriodaphnia dubia*) [50]																					
Sarafloxacin		4.6	<1	3.6	<1	1.0	<1	<1	<1	1.9	<1	<1	NA	<1	<1	<1	NA	NA	<1	<1	<1	NA
PNEC	1.87 [30]																					
EC_50_	15 (*Microcystis aeruginosa*) [51]																					
Furosemide		<1	<1	<1	<1	<1	<1	<1	<1	<1	<1	<1	<1	<1	<1	NA	<1	<1	<1	<1	NA	NA
PNEC	31.3 [30]																					
EC_50_	60,600 (*Dapnia magna*) [52]																					
Topiramate		<1	<1	<1	<1	<1	<1	<1	<1	<1	<1	<1	<1	<1	<1	<1	<1	<1	<1	<1	<1	<1
PNEC	930 [30]																					
LC_50_	230,086 (*Danio rerio*) [53]																					
EC_50_	293,037 (*Danio rerio*) [53]																					
Valsartan		<1	<1	<1	<1	<1	<1	<1	<1	<1	<1	<1	<1	<1	<1	NA	<1	<1	<1	<1	<1	<1
PNEC	560 [30]																					
LC_50_	49,000 (fish), 27,000 (crustaceans) [54]																					
EC_50_	14,000 (Algae) [54]																					

^a^ Not available.

## Data Availability

Data are contained within the article and Supplementary Materials.

## Supplementary Materials

The following supporting information can be downloaded at: https://www.mdpi.com/article/10.3390/molecules31030455/s1, Figure S1: Representative LC-MS/MS chromatogram of the mixture of standards solution of the 70 analyzed pharmaceuticals; Figure S2: Representative LC-MS/MS chromatograms of environmental water sample from Cerrón Grande reservoir ID2 from (A) summer season and (B) rainy season; Table S1: Quality parameters of pharmaceuticals. Values are expressed in μg L^−1^. LOD and LOQ represent values in matrix; Table S2: Concentration of pharmaceuticals in the 12 sampling points in the dry season. Values are expressed in μg L^−1^; Table S3: Concentration of pharmaceuticals in the 9 sampling points in the rainy season. Values are expressed in μg L^−1^.
