# Supplementary material for: First Evidence of Pharmaceutical Residues in the Cerrón Grande Reservoir, El Salvador
Source: Molecules. 2026 Jan 28;31(3):455. doi: 10.3390/molecules31030455 (PMC12898499; doi:10.3390/molecules31030455)
Supplement: Supplementary file 1 [file molecules-31-00455-s001.zip › molecules-4079198-supplementary.pdf]

Supplementary material

Table S1. Quality parameters of pharmaceuticals. Values are expressed in μg L^{-1}. LOD and LOQ represent values in matrix.

**Pharmaceuticals**	**CAS Number**	Linearity range	LOD	LOQ	Recovery (%)	Matrix effect (%)
1,7-Dimethylxanthine	611-59-6	5 – 0.01	0.015	0.050	105	109
4-acetamidoantipyrine	83-15-8	1 – 0.01	0.020	0.066	90	92
4-aminoantipyrine	83-07-8	5 – 0.001	0.001	0.003	104	95
Acetaminophen	103-90-2	5 – 0.001	0.007	0.023	75	71
Acyclovir	59277-89-3	1 – 0.001	0.005	0.018	107	75
Amiodarone	1951-25-3	1 – 0.001	0.010	0.033	60	78
Amitriptyline	50-48-6	5 – 0.001	0.001	0.003	105	97
Antipyrine	60-80-0	1 – 0.001	0.001	0.003	94	98
Atorvastatin	134523-00-5	1 – 0.001	0.001	0.003	114	94
Bicalutamide	90357-06-5	5 – 0.001	0.001	0.003	133	102
Bisoprolol	66722-44-9	5 – 0.001	0.001	0.003	100	86
Caffeine	58-08-2	5 – 0.001	0.010	0.033	107	81
Capecitabine	154361-50-9	5 – 0.001	0.001	0.003	84	74
Chlormethiazole	533-45-9	5 – 0.001	0.008	0.026	102	79
Chloroquine	54-05-7	5 – 0.001	0.017	0.055	87	102
Chlorpromazine	50-53-3	5 – 0.001	0.003	0.008	94	84
Cimetidine	51481-61-9	1 – 0.001	0.008	0.026	130	81
Citalopram	59729-33-8	5 – 0.001	0.001	0.003	123	105
Clopidogrel	113665-84-2	1 – 0.001	0.002	0.007	67	80
Cyclophosphamide	6055-19-2	1 – 0.001	0.001	0.003	137	108
Desloratadine	100643-71-8	5 – 0.001	0.005	0.017	121	112
Dexamethasone	50-02-2	1 – 0.001	0.001	0.003	105	89
Diclofenac	15307-86-5	1 – 0.001	0.001	0.003	103	82
Diflubenzuron	35367-38-5	1 – 0.001	0.001	0.003	86	104
Fenofibrate	49562-28-9	5 – 0.001	0.002	0.006	98	95
Fexofenadine	885946-90-7	5 – 0.001	0.002	0.005	111	109
Fluoxetine	54910-89-3	5 – 0.001	0.002	0.005	91	94

Fluticasone	90566-53-3	5 – 0.001	0.002	0.005	106	108
Fluvoxamine	54739-18-3	5 – 0.001	0.002	0.005	98	86
Furosemide	54-31-9	5 – 0.001	0.004	0.012	82	109
Gliclazide	21187-98-4	5 – 0.001	0.003	0.010	76	90
Hydroxychloroquine	118-42-3	5 – 0.01	0.100	0.330	92	100
Ifosfamide	3778-73-2	1 – 0.001	0.005	0.017	95	71
Ivermectin	70288-86-7	5 – 0.001	0.002	0.005	120	116
Lamotrigine	84057-84-1	1 – 0.001	0.001	0.003	87	104
Levetiracetam	102767-28-2	1 – 0.001	0.002	0.005	131	107
Levofloxacin	100986-85-4	5 – 0.001	0.002	0.005	120	102
Lidocaine	137-58-6	5 – 0.001	0.002	0.006	113	124
Lopinavir	192725-17-0	5 – 0.001	0.002	0.005	103	118
Levetiracetam	102767-28-2	5 – 0.001	0.002	0.005	132	108
Manidipine	89226-50-6	5 – 0.001	0.030	0.099	95	106
Mecamylamine	60-40-2	5 – 0.001	0.020	0.066	114	110
Metformin	1115-70-4	1 – 0.001	0.030	0.099	104	107
Metoprolol	51384-51-1	5 – 0.001	0.004	0.013	127	103
Mevinolin	75330-75-5	5 – 0.01	0.020	0.066	98	109
Molnupiravir	2492423-29-5	5 – 0.001	0.017	0.055	110	94
Mycophenolic acid	24280-93-1	5 – 0.001	0.003	0.008	116	101
Norfloxacin	70458-96-7	5 – 0.001	0.002	0.007	135	114
Nortriptyline	72-69-5	5 – 0.001	0.001	0.003	115	106
Omeprazole	73590-58-6	5 – 0.001	0.017	0.055	127	100
Oxytetracycline	79-57-2	5 – 0.001	0.017	0.055	78	80
Pentoxifylline	6493-05-6	5 – 0.001	0.001	0.003	116	125
Propranolol	525-66-6	1 – 0.001	0.001	0.003	115	94
Quetiapine	111974-69-7	1 – 0.001	0.001	0.003	123	88
Ranitidine	66357-35-5	5 – 0.001	0.001	0.003	102	110
Ranolazine	95635-55-5	1 – 0.001	0.001	0.003	79	77
Reboxetine	71620-89-8	5 – 0.001	0.001	0.003	94	72

Remdesivir	1809249-37-3	5 – 0.001	0.003	0.010	93	89
Ritonavir	155213-67-5	1 – 0.001	0.001	0.003	126	121
Rosuvastatin	287714-41-4	1 – 0.001	0.004	0.013	99	106
Rotigotine	99755-59-6	5 – 0.001	0.002	0.007	114	97
Sarafloxacin	98105-99-8	1 – 0.001	0.001	0.003	102	105
Scopolamine	51-34-3	5 – 0.001	0.001	0.003	114	111
Sitagliptin	486460-32-6	5 – 0.001	0.001	0.003	81	101
Sulfadiazine	68-35-9	1 – 0.001	0.001	0.003	130	107
Sulfamethoxazole	723-46-6	5 – 0.001	0.007	0.022	79	71
Sulfapyridine	144-83-2	5 – 0.001	0.001	0.003	99	78
Tetracycline	60-54-8	5 – 0.001	0.010	0.033	104	97
Topiramate	97240-79-4	5 – 0.001	0.002	0.007	94	87
Trazodone	19794-93-5	0.5 – 0.001	0.001	0.003	103	91
Trimethoprim	738-70-5	1 – 0.001	0.002	0.007	101	83
Valsartan	137862-53-4	5 – 0.005	0.007	0.023	98	96
Venlafaxine	93413-69-5	1 – 0.001	0.003	0.010	136	101
Verapamil	52-53-9	1 – 0.001	0.005	0.017	94	116
Vildagliptin	274901-16-5	5 – 0.001	0.002	0.007	125	121
Zuclopenthixol	53772-83-1	5 – 0.001	0.008	0.026	112	90

Table S2. Concentration of pharmaceuticals in the 12 sampling points in the dry season. Values are expressed in $\mu g\ L^{-1}$.

**Pharmaceuticals**	ID 1	ID 2	ID 3	ID 4	ID 5	ID 6	ID 7	ID 8	ID 9	ID 10	ID 11	ID 12
1,7-Dimethylxanthine	529.4	563.2	954.7	561.4	1005.2	1069.8	1121.1	1084.8	2829.0	1540.6	1907.1	379.7
4-acetamidoantipyrine	5.50	5.27	5.907	5.638	6.47	5.86	7.19	7.70	7.00	8.00	9.62	1.97
4-aminoantipyrine	0.305	<LOD	<LOD	<LOD	<LOD	<LOD	<LOD	<LOD	<LOD	<LOD	<LOD	<LOD
Acetaminophen	51.6	18.6	120.6	11.7	25.0	38.7	28.1	10.2	39.8	15.5	19.5	<LOD
Acyclovir	1.71	1.41	2.50	2.83	2.61	3.22	2.76	3.52	3.572	3.02	3.48	1.51
Amiodarone	0.324	0.317	0.323	0.289	2.69	0.386	0.358	0.510	0.278	0.294	0.286	0.302
Amitriptyline	2.03	0.568	1.09	0.734	9.89	0.984	0.837	0.673	1.42	0.611	1.14	0.195
Antipyrine	0.323	0.205	0.322	0.338	0.270	0.300	0.236	0.311	0.335	0.362	0.307	0.345
Atorvastatin	0.081	0.053	0.081	0.056	0.091	0.057	0.057	0.083	0.064	0.056	0.088	0.045
Bicalutamide	14.4	6.63	22.6	11.4	8.58	8.85	5.68	4.74	10.9	6.75	6.06	0.799
Bisoprolol	0.581	0.186	1.77	1.190	21.0	1.34	5.58	3.07	1.61	1.16	8.37	0.184
Caffeine	27.2	29.9	50.5	39.1	35.4	27.5	29.0	21.4	44.3	22.4	35.2	17.8
Capecitabine	0.315	0.113	0.200	0.076	0.364	0.095	0.103	0.116	0.172	0.222	0.139	0.043
Chlormethiazole	0.557	0.175	0.283	0.155	0.205	0.189	0.174	0.183	0.173	0.175	0.188	0.170
Chloroquine	322.2	2.76	224.9	2.29	64.4	19.0	3.17	2.78	362.7	4.44	32.9	2.52
Chlorpromazine	0.278	<LOD	0.225	0.039	0.180	0.188	0.050	0.054	0.305	0.057	0.085	0.038
Cimetidine	1.53	0.550	1.63	1.35	0.876	1.59	1.25	3.35	1.44	3.05	1.78	2.17
Citalopram	1.75	0.479	1.47	0.446	1.30	0.358	0.943	0.438	0.853	0.401	0.727	0.113
Clopidogrel	0.026	<LOD	0.049	<LOD	<LOD	0.008	0.081	<LOD	0.068	<LOD	0.057	0.008
Cyclophosphamide	0.048	0.014	0.028	0.011	0.018	0.009	0.027	0.013	0.028	0.044	0.020	0.009
Desloratadine	2.63	0.230	3.13	0.276	0.647	0.235	0.251	0.211	<LOD	<LOD	<LOD	<LOD
Dexamethasone	<LOD	<LOD	<LOD	<LOD	<LOD	<LOD	<LOD	<LOD	<LOD	<LOD	<LOD	<LOD
Diclofenac	0.807	0.454	0.731	0.324	0.361	1.32	0.912	2.95	<LOD	<LOD	<LOD	<LOD
Diflubenzuron	0.017	0.031	0.021	0.022	0.705	0.240	0.043	0.019	0.030	0.057	0.017	0.018
Fenofibrate	0.061	0.119	0.074	0.037	1.27	0.645	0.043	0.028	0.048	0.146	0.016	0.074
Fexofenadine	11.9	7.80	18.2	13.6	17.2	17.0	19.4	21.5	21.0	23.7	27.2	17.1
Fluoxetine	1.70	0.334	1.52	0.549	18.1	0.749	0.560	0.503	3.056	0.682	1.55	0.136
Fluticasone	<LOD	<LOD	<LOD	<LOD	<LOD	<LOD	<LOD	<LOD	<LOD	<LOD	<LOD	<LOD

Fluvoxamine	0.681	0.529	0.422	0.324	3.43	0.388	<LOD	<LOD	1.34	<LOD	<LOD	<LOD
Furosemide	11.0	8.25	12.5	8.02	7.38	7.09	4.18	6.54	7.76	9.28	5.82	1.31
Gliclazide	0.011	0.038	0.017	0.012	0.013	0.016	0.014	<LOD	0.040	0.014	0.025	0.011
Hydroxychloroquine	315.4	9.95	189.7	8.91	89.9	12.6	7.85	8.36	204.8	5.02	16.4	6.23
Ifosfamide	<LOD	<LOD	0.231	0.136	0.363	0.723	0.097	0.138	0.318	<LOD	0.455	<LOD
Ivermectin	8.39	0.744	13.5	0.477	20.9	0.218	9.17	8.23	2.55	1.83	0.966	0.198
Lamotrigine	0.685	0.739	0.792	0.807	0.905	0.731	0.608	0.950	0.826	0.769	0.614	0.243
Levetiracetam	0.498	0.357	0.415	0.365	0.261	0.272	0.325	0.287	0.168	0.665	0.616	<LOD
Levofloxacin	16.4	19.8	17.0	3.87	34.2	3.32	3.53	1.23	10.7	3.01	2.87	0.080
Lidocaine	2.05	0.868	1.80	1.21	2.20	0.916	1.77	1.27	2.13	1.55	2.42	0.549
Lopinavir	0.875	0.215	1.00	0.054	0.784	0.115	0.063	0.095	0.774	0.095	0.104	0.046
Levetiracetam	0.195	0.110	0.119	0.148	0.098	0.109	0.130	0.108	0.078	0.264	0.201	<LOD
Manidipine	1.12	<LOD	1.23	0.919	0.989	<LOD	0.958	1.04	0.980	0.963	0.957	0.924
Mecamylamine	6218.0	4765.5	6913.2	5315.8	6874.5	5415.9	4166.8	5063.2	3822.0	5015.8	5452.4	1710.8
Metformin	6.22	<LOD	7.71	7.57	6.81	8.57	7.98	14.3	7.51	6.30	9.67	14.8
Metoprolol	0.512	0.282	0.528	0.753	1.36	0.474	0.269	0.724	0.850	0.344	0.356	0.199
Mevinolin	6.19	26.3	16.4	11.3	24.9	11.1	6.21	8.96	10.1	7.29	5.41	2.69
Molnupiravir	2.23	1.129	1.42	4.95	2.91	1.26	1.32	1.48	1.94	4.02	3.43	<LOD
Mycophenolic acid	<LOD	<LOD	<LOD	<LOD	<LOD	<LOD	<LOD	<LOD	<LOD	<LOD	<LOD	<LOD
Norfloxacin	68.3	9.252	66.2	12.1	34.3	2.99	2.88	0.784	37.5	2.14	4.67	<LOD
Nortriptyline	0.662	0.066	0.540	0.151	1.13	0.079	0.071	0.070	1.19	0.061	0.212	0.012
Omeprazole	0.794	0.727	0.757	0.657	0.717	0.700	0.658	0.740	0.788	0.778	0.678	0.612
Oxytetracycline	6.51	6.31	3.34	4.01	11.1	<LOD	<LOD	<LOD	<LOD	2.05	12.5	<LOD
Pentoxifylline	0.037	0.019	0.024	0.017	0.039	<LOD	0.024	0.026	0.024	<LOD	0.025	0.012
Propranolol	0.098	0.054	0.114	0.054	0.317	0.048	0.022	<LOD	0.179	<LOD	0.041	0.037
Quetiapine	0.970	0.031	1.36	0.068	0.630	0.075	0.064	0.066	0.554	0.058	0.238	0.067
Ranitidine	<LOD	<LOD	<LOD	<LOD	<LOD	<LOD	<LOD	<LOD	<LOD	<LOD	<LOD	<LOD
Ranolazine	0.084	0.078	0.090	0.089	0.101	0.069	0.153	0.119	0.086	0.093	0.170	0.135
Reboxetine	<LOD	<LOD	<LOD	<LOD	<LOD	0.044	0.183	0.006	0.025	0.049	0.316	0.034
Remdesivir	1.28	1.11	1.12	1.081	1.33	1.07	1.53	1.02	1.04	1.06	<LOD	1.02

Ritonavir	0.527	0.159	0.369	0.088	0.743	0.099	0.069	0.083	0.305	0.086	0.057	0.025
Rosuvastatin	0.153	0.137	0.134	0.118	0.375	0.105	0.114	0.113	0.153	0.112	0.118	0.085
Rotigotine	0.808	<LOD	0.723	0.130	0.725	<LOD	<LOD	<LOD	0.901	0.317	0.626	<LOD
Sarafloxacin	8.57	0.416	6.78	1.42	1.87	0.082	0.169	0.098	3.57	0.161	0.715	<LOD
Scopolamine	0.305	0.121	0.695	0.094	0.335	0.065	0.088	0.078	0.427	0.082	0.113	0.022
Sitagliptin	0.239	0.194	0.180	0.071	0.187	0.073	0.122	0.102	0.108	0.075	0.134	0.048
Sulfadiazine	0.014	<LOD	0.006	0.005	0.004	<LOD	<LOD	0.004	0.002	0.003	0.005	0.001
Sulfamethoxazole	5.57	1.69	4.98	4.74	2.43	4.83	4.26	14.1	4.38	12.0	6.16	5.35
Sulfapyridine	0.060	0.014	0.034	0.114	0.033	0.009	0.013	0.088	0.012	0.017	0.038	0.012
Tetracycline	1.40	<LOD	0.928	1.54	2.60	0.543	0.327	<LOD	0.443	0.434	1.24	0.293
Topiramate	3.26	0.510	1.99	1.77	3.06	1.74	2.22	2.67	2.89	2.86	1.51	2.17
Trazodone	0.028	<LOD	0.041	<LOD	0.027	0.026	0.075	<LOD	0.066	<LOD	0.018	0.006
Trimethoprim	0.740	0.525	0.562	0.528	0.819	0.613	0.519	0.727	0.807	0.902	1.16	0.474
Valsartan	29.2	52.2	38.9	35.9	27.7	38.2	31.8	63.2	25.7	81.6	86.3	9.01
Venlafaxine	0.664	0.250	0.562	0.317	1.10	0.295	0.416	0.321	0.696	0.304	0.406	0.192
Verapamil	0.938	0.238	1.07	0.215	4.23	0.218	0.249	0.235	1.79	0.204	0.353	0.088
Vildagliptin	0.331	0.370	0.435	0.406	0.459	0.319	0.510	0.444	0.466	0.686	0.560	<LOD
Zuclopenthixol	3.10	0.819	2.42	<LOD	1.29	<LOD	<LOD	<LOD	2.44	<LOD	1.10	0.841

Table S3. Concentration of pharmaceuticals in the 9 sampling points in the rainy season. Values are expressed in μg L^{-1}.

**Pharmaceuticals**	ID 1	ID 2	ID 3	ID 4	ID 5	ID 6	ID 7	ID 8	ID 9
1,7-Dimethylxanthine	0.018	0.4394	0.4167	0.1197	0.2936	0.4737	0.5031	0.5547	0.0757
4-acetamidoantipyrine	0.026	0.0242	0.0252	0.0218	0.0254	0.0250	0.0258	0.0254	0.0232
4-aminoantipyrine	<LOD	<LOD	<LOD	<LOD	<LOD	<LOD	<LOD	<LOD	<LOD
Acetaminophen	0.017	0.0426	0.0462	0.0088	0.0297	0.0482	0.0357	0.0226	0.0199
Acyclovir	0.006	0.0075	0.0105	0.0080	0.0105	0.0134	0.0141	0.0154	0.0140
Amiodarone	0.028	0.0271	0.0277	0.0275	<LOD	0.0270	0.0272	0.0275	0.0272
Amitriptyline	<LOD	0.0012	<LOD	<LOD	<LOD	<LOD	<LOD	<LOD	<LOD
Antipyrine	0.003	0.0045	0.0043	0.0047	0.0040	0.0037	0.0040	0.0046	0.0041
Atorvastatin	0.005	0.0044	0.0043	0.0041	0.0042	0.0041	0.0041	0.0041	0.0041
Bicalutamide	<LOD	0.0014	0.0034	<LOD	0.0022	<LOD	<LOD	0.0013	0.0021
Bisoprolol	<LOD	<LOD	<LOD	<LOD	<LOD	<LOD	<LOD	<LOD	<LOD
Caffeine	0.242	0.4737	0.2107	0.1996	0.2738	0.2608	0.3417	0.2202	0.3532
Capecitabine	<LOD	<LOD	<LOD	<LOD	<LOD	<LOD	<LOD	<LOD	<LOD
Chlormethiazole	0.019	0.0169	0.0157	0.0174	0.0161	0.0169	0.0157	0.0155	0.0174
Chloroquine	<LOD	<LOD	<LOD	<LOD	<LOD	<LOD	<LOD	<LOD	<LOD
Chlorpromazine	0.0034	0.0032	<LOD	0.0031	<LOD	0.0031	0.0031	<LOD	0.0031
Cimetidine	0.023	0.0183	0.0204	0.0248	0.0223	0.0230	0.0272	0.0222	0.0292
Citalopram	<LOD	<LOD	<LOD	<LOD	<LOD	<LOD	<LOD	<LOD	<LOD
Clopidogrel	<LOD	<LOD	<LOD	<LOD	<LOD	<LOD	<LOD	<LOD	<LOD
Cyclophosphamide	<LOD	<LOD	<LOD	<LOD	<LOD	<LOD	<LOD	<LOD	<LOD
Desloratadine	0.015	<LOD	0.015	0.015	0.015	0.015	0.015	0.015	<LOD
Dexamethasone	0.003	0.0017	0.0011	0.0016	0.0027	<LOD	0.0018	0.0015	0.0020
Diclofenac	0.008	0.0043	0.0012	0.0017	<LOD	0.0064	0.0061	0.0023	0.0036
Diflubenzuron	0.001	<LOD	<LOD	<LOD	<LOD	<LOD	<LOD	n.d	n.d
Fenofibrate	0.0025	<LOD	<LOD	<LOD	<LOD	<LOD	<LOD	<LOD	<LOD
Fexofenadine	0.0023	0.0078	0.0041	0.0050	0.0053	0.0043	0.0049	0.0061	0.0063
Fluoxetine	<LOD	<LOD	<LOD	<LOD	<LOD	<LOD	<LOD	<LOD	<LOD
Fluticasone	<LOD	0.0022	0.0020	<LOD	<LOD	<LOD	<LOD	<LOD	<LOD

Fluvoxamine	0.005	0.0039	<LOD	0.0036	0.0033	0.0034	<LOD	<LOD	<LOD
Furosemide	0.010	0.0177	<LOD	0.0128	0.0097	0.0219	0.0092	<LOD	<LOD
Gliclazide	<LOD	<LOD	<LOD	<LOD	<LOD	<LOD	<LOD	<LOD	<LOD
Hydroxychloroquine	<LOD	<LOD	<LOD	<LOD	<LOD	<LOD	<LOD	<LOD	<LOD
Ifosfamide	0.015	0.0248	0.0283	0.0323	0.0151	0.0214	0.0224	0.0246	0.0247
Ivermectin	<LOD	0.0022	<LOD	<LOD	<LOD	<LOD	<LOD	<LOD	<LOD
Lamotrigine	0.0054	0.0052	0.0047	0.0043	0.0054	0.0055	0.0042	0.0049	0.0055
Levetiracetam	0.0038	0.0036	0.0040	0.0032	0.0040	0.0036	0.0035	0.0027	0.0036
Levofloxacin	0.0021	<LOD	<LOD	<LOD	<LOD	<LOD	<LOD	<LOD	<LOD
Lidocaine	0.0062	0.0051	0.0057	0.0055	0.0073	0.0070	0.0063	0.0066	0.0072
Lopinavir	<LOD	<LOD	<LOD	<LOD	<LOD	<LOD	<LOD	<LOD	<LOD
Levetiracetam	0.0044	0.0030	0.0039	0.0028	0.0044	0.0034	0.0037	0.0047	0.0039
Manidipine	<LOD	0.0907	0.0908	<LOD	0.0907	<LOD	0.0907	<LOD	0.0908
Mecamylamine	1635.7	1917.3	1667.9002	1795.1	1525.6	1784.9	1606.6	1772.1	2198.1
Metformin	<LOD	<LOD	<LOD	<LOD	0.1418	<LOD	<LOD	<LOD	<LOD
Metoprolol	0.0060	<LOD	<LOD	<LOD	<LOD	<LOD	<LOD	<LOD	<LOD
Mevinolin	<LOD	0.036	<LOD	0.076	0.073	<LOD	0.026	0.046	<LOD
Molnupiravir	<LOD	0.082	0.081	0.082	<LOD	0.081	0.081	<LOD	0.083
Mycophenolic acid	0.0057	0.0060	0.0039	0.0065	0.0054	0.0042	0.0044	0.0058	0.015
Norfloxacin	<LOD	0.0036	0.0034	0.0020	<LOD	<LOD	<LOD	<LOD	<LOD
Nortriptyline	<LOD	<LOD	<LOD	<LOD	<LOD	<LOD	<LOD	<LOD	<LOD
Omeprazole	0.061	0.061	0.061	0.060	0.061	<LOD	0.061	<LOD	0.062
Oxytetracycline	<LOD	0.2180	0.0785	0.1967	0.0662	0.0725	0.0621	<LOD	<LOD
Pentoxifylline	<LOD	<LOD	<LOD	<LOD	<LOD	<LOD	<LOD	<LOD	<LOD
Propranolol	<LOD	<LOD	<LOD	<LOD	<LOD	<LOD	<LOD	<LOD	<LOD
Quetiapine	<LOD	<LOD	<LOD	<LOD	<LOD	<LOD	<LOD	<LOD	<LOD
Ranitidine	<LOD	<LOD	<LOD	<LOD	<LOD	<LOD	<LOD	<LOD	<LOD
Ranolazine	<LOD	<LOD	<LOD	<LOD	<LOD	<LOD	<LOD	<LOD	<LOD
Reboxetine	<LOD	<LOD	<LOD	<LOD	<LOD	<LOD	<LOD	<LOD	<LOD
Remdesivir	0.100	0.100	<LOD	<LOD	<LOD	<LOD	<LOD	0.101	<LOD

Ritonavir	0.0015	<LOD	<LOD	<LOD	<LOD	<LOD	<LOD	<LOD	<LOD
Rosuvastatin	0.0079	0.0078	0.0078	<LOD	<LOD	0.0078	0.0078	<LOD	<LOD
Rotigotine	0.0117	0.0070	0.0047	0.0026	0.0097	0.0060	0.0059	0.0088	0.0128
Sarafloxacin	0.0012	<LOD	<LOD	<LOD	<LOD	<LOD	<LOD	<LOD	<LOD
Scopolamine	<LOD	<LOD	<LOD	<LOD	<LOD	n.d	<LOD	<LOD	<LOD
Sitagliptin	0.0011	<LOD	<LOD	<LOD	<LOD	<LOD	<LOD	<LOD	<LOD
Sulfadiazine	0.0015	<LOD	<LOD	<LOD	<LOD	<LOD	<LOD	<LOD	<LOD
Sulfamethoxazole	0.0379	0.0303	0.0306	0.0303	0.0356	0.0338	0.0313	0.0305	0.0350
Sulfapyridine	<LOD	<LOD	<LOD	<LOD	<LOD	<LOD	<LOD	<LOD	<LOD
Tetracycline	0.0270	0.0231	0.0232	<LOD	<LOD	0.0233	<LOD	0.0231	<LOD
Topiramate	0.0165	0.0221	0.0055	0.0221	0.0055	0.0108	0.0275	0.0111	0.0055
Trazodone	<LOD	<LOD	n.d	n.d	<LOD	<LOD	n.d	n.d	n.d
Trimethoprim	0.0023	0.0023	0.0025	0.0024	0.0027	0.0028	0.0024	0.0031	0.0026
Valsartan	0.0276	0.0320	<LOD	0.0197	0.0721	0.0470	0.0191	0.0139	0.0249
Venlafaxine	<LOD	0.0144	0.0144	0.0144	0.0144	0.0144	0.0144	0.0144	0.0163
Verapamil	0.0058	0.0060	0.0059	0.0058	0.0059	0.0059	<LOD	0.0062	0.0059
Vildagliptin	0.0033	0.0037	0.0042	0.0038	0.0049	0.0028	0.0042	0.0044	0.0058
Zuclopenthixol	0.0797	0.0790	0.0788	0.0788	0.0788	<LOD	0.0788	0.0791	0.0788

*n.d are not detected compounds

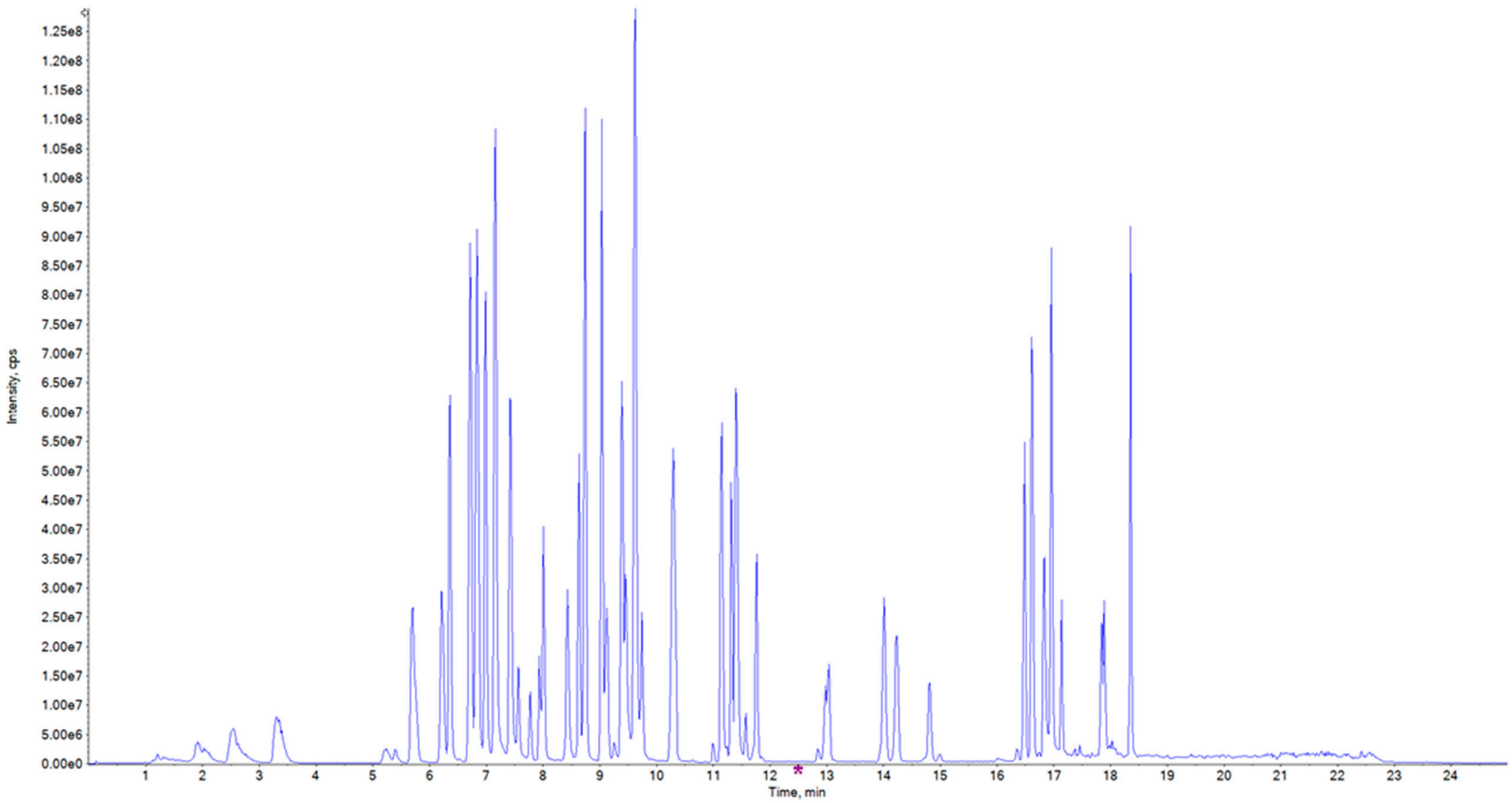

Figure S1. Representative LC-MS/MS chromatogram of the mixture of standards solution of the 70 analyzed pharmaceuticals.

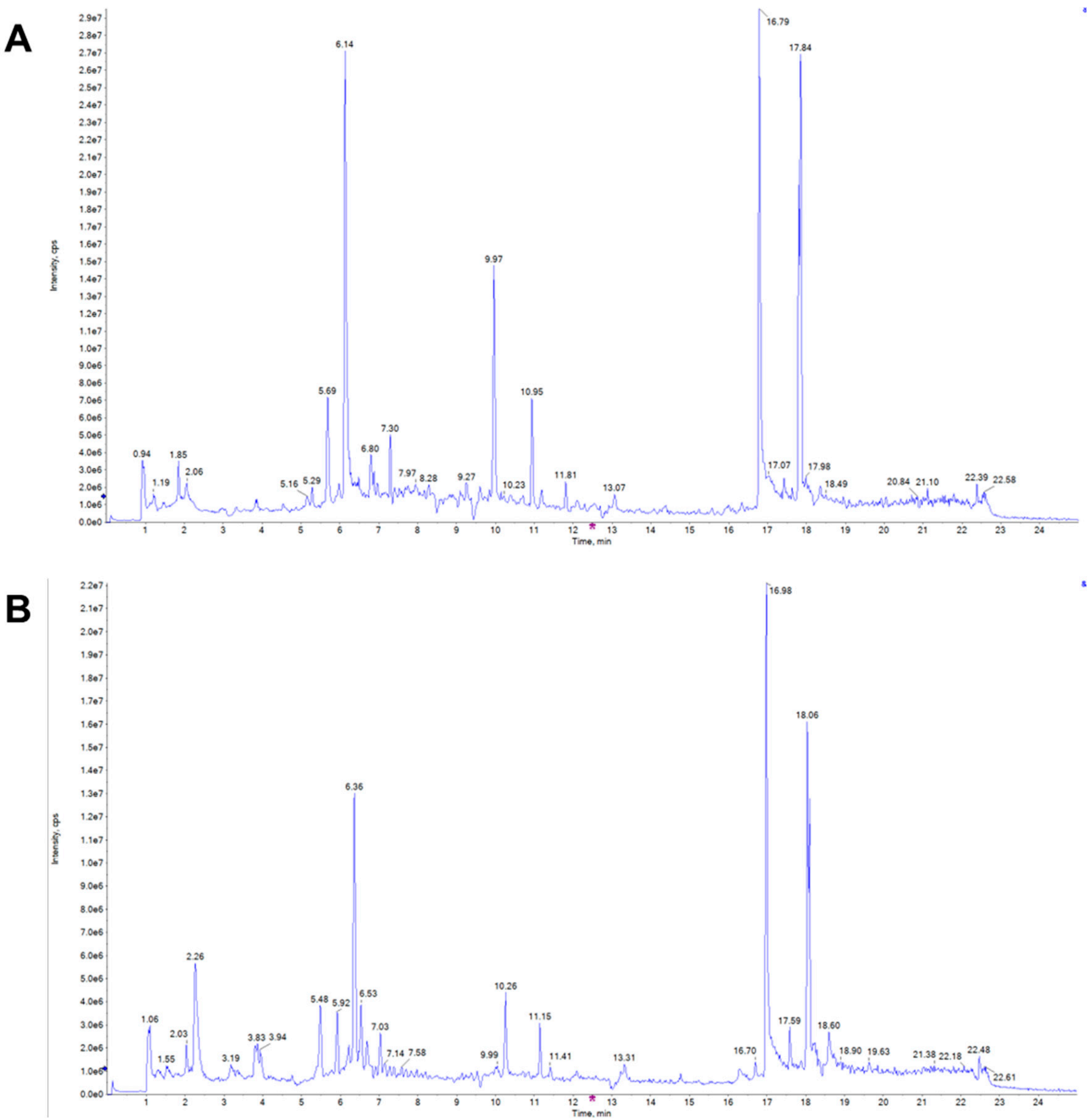

Figure S2. Representative LC-MS/MS chromatograms of environmental water sample from Cerrón Grande reservoir ID2 from (A) summer season and (B) rainy season.
